# Resection of oesophageal and oesophagogastric junction cancer liver metastases — a summary of current evidence

**DOI:** 10.1007/s00423-021-02387-3

**Published:** 2021-12-03

**Authors:** Andreas R. R. Weiss, Noel E. Donlon, Hans J. Schlitt, Christina Hackl

**Affiliations:** 1 Department of Surgery, University Hospital Regensburg, Franz-Josef-Strauss-Allee 11, 93053 Regensburg, Germany; 2 Trinity Translational Medicine Institute, Department of Surgery, Trinity College Dublin, St. James’s Hospital, Dublin 8, Dublin, Ireland

**Keywords:** Oesophageal cancer, Liver metastases, Oesophagogastric junction cancer, Liver resection, Metastasectomy, Multimodal treatment

## Abstract

**Purpose:**

Metastatic oesophageal cancer is commonly considered as a palliative situation with a poor prognosis. However, there is increasing evidence that well-selected patients with a limited number of liver metastases (ECLM) may benefit from a multimodal approach including surgery.

**Methods:**

A systematic review of the current literature for randomized trials, retrospective studies, and case series with patients undergoing hepatectomies for oesophageal and oesophagogastric junction cancer liver metastases was conducted up to the 31st of August 2021 using the MEDLINE (PubMed) and Cochrane Library databases.

**Results:**

A total of 661 articles were identified. After removal of duplicates, 483 articles were screened, of which 11 met the inclusion criteria. The available literature suggests that ECLM resection in patients with liver oligometastatic disease may lead to improved survival and even long-term survival in some cases. The response to concomitant chemotherapy and liver resection seems to be of significance. Furthermore, a long disease-free interval in metachronous disease, low number of liver metastases, young age, and good overall performance status have been described as potential predictive markers of outcome for the resection of liver metastases.

**Conclusion:**

Surgery may be offered to carefully selected patients to potentially improve survival rates compared to palliative treatment approaches. Studies with standardized patient selection criteria and treatment protocols are required to further define the role for surgery in ECLM. In this context, particular consideration should be given to neoadjuvant treatment concepts including immunotherapies in stage IVB oesophageal and oesophagogastric junction cancer.

## Introduction

The treatment of oesophageal cancer has markedly improved over the last decades. Embedded in a multimodal treatment concept, 5-year survival rates around 50% can be achieved given the absence of distant metastatic disease [1, 2]. However, patients with stage IVB oesophageal cancer are generally treated in a palliative intention with chemotherapy or best supportive care and have a poor median survival between 4 and 8 months [3].

Hepatic metastases remain one of the most common sites of distant dissemination in oesophageal cancer (EC) with an incidence of 35–40% at the time of diagnosis. It also represents the first site of recurrence in 6–25% of cases after oesophagectomy with curative intent [4, 5].

Over the last decades, liver resections for metastatic cancer have become a standard treatment for defined tumour entities, especially in patients with colorectal cancer (CRC) and neuroendocrine tumours. Overall perioperative mortality rates between 1 and 5% have been reported, although a high dependency on case load per centre and the average extent of liver surgeries (minor resections up to extended hepatectomies) have to be taken into consideration when comparing these studies [6–8]. However, 5-year overall survival (OS) rates over 50% was achievable in patients who underwent liver resections for CRC liver metastases [9, 10].

The role for liver resections in liver oligometastatic EC remains controversial and the available literature is scarce. Some studies reported improved survival rates in patients, who underwent oesophageal cancer liver metastases (ECLM) resection, if compared to palliative treatment alone [11–13]. Other studies reported little or no survival benefit, which raises the question what criteria might help to stratify patients that may benefit from a surgical approach [14, 15]. This article provides an overview of the available literature and the role for surgery in patients with ECLM.

## Methods

A systematic literature search was conducted using the MEDLINE (PubMed) and Cochrane Library databases. Studies with cancer patients undergoing surgery for oesophageal or gastroesophageal cancer liver metastases were included, if an appropriate follow-up with information on survival following surgery was available. The search strategy included combinations of the following keywords: ‘esophageal cancer’, ‘esophagogastric junction cancer’, ‘gastroesophageal adenocarcinoma’, ‘liver resection’, ‘hepatectomy’, and ‘metastasectomy’.

Only original articles in English were considered for this systematic review. Case reports and case series with less than 4 patients were excluded. Studies with patients, who underwent liver resections for multiple tumour entities, were excluded, if there was no sufficient subgroup data available on patients with oesophageal or oesophagogastric junction cancer liver metastases.

The search was carried out until the 31st of August 2021 and identified 653 articles. Further 8 articles were added from the references of other systematic reviews. After removal of duplicates, 483 articles were screened. Following this, 407 articles were excluded as abstracts and titles did not meet the inclusion criteria. The remaining 76 articles were reviewed by full text. Finally, 11 articles met the inclusion criteria and were included in the systematic review.

## Resection for non-colorectal/non-neuroendocrine liver metastases

The surgical treatment for colorectal and neuroendocrine liver metastases has become the standard of care, given that a complete curative resection can be achieved [16, 17]. However, liver metastasis resection for non-colorectal/non-neuroendocrine primary tumours remains a controversial issue and no clear international level 1 recommendations are available, with many basing practice on case series and anecdotal evidence. Some studies demonstrate improved short-term survival rates and even long-term survival in patients, who underwent resections for non-colorectal/non-neuroendocrine liver metastases (NCNNLM) [18–21]. Following the resection of NCNNLM, 5-year overall survival rates between 30 and 61% have been reported [18, 20, 21]. However, direct correlations on reported survival rates cannot be drawn due to heterogeneity of primary tumours and the percentage of patients with ECLM within these studies is generally very low to zero. It has been demonstrated that the survival advantage depends on the primary site with patients undergoing resection for genitourinary liver metastases benefitting the most in terms of overall survival [22]. Patients with liver metastases from gastroesophageal primaries were found to have less favourable results with a median survival between 16 and 26 months [18, 22, 23].

However, a recent study from 2018 analysed 1792 patients with metastatic adenocarcinoma of the stomach, the oesophagogastric junction, and the distal oesophagus [24]. Of these, 92 patients (5%) underwent metastasectomy with the most common metastatic sites being peritoneal (29%), hepatic (24%), and distant lymph nodes (11%). The study included two patients with oesophageal and ten patients with oesophagogastric junction primaries. Patients, who underwent surgery for metastatic disease, had higher survival rates compared to conservatively treated patients. The median OS after metastasectomy was 16.7 months with a 3-year OS after surgery of 30.6%, which is beyond the results that can be achieved with palliative chemotherapy alone [3].

Similar results were provided by Schmidt et al., who reported a median survival of 21.3 months with a 3-year OS of 29.5% and a 5-year OS of 21.9% in 112 patients with metastatic gastric or oesophagogastric junction cancer that underwent surgical resection [25]. Badgwell et al. demonstrated a 5-year OS of 25% in 82 patients after resection of oesophagogastric cancer metastases [26]. The subgroup of patients, in whom solid organ metastases were resected, had an even higher 5-year OS of 34%. Another study by Andreou et al. reported a median survival of 18 months in 47 patients undergoing hepatectomy for oesophagogastric cancer metastases with 3- and 5-year OS rates of 37% and 24%, respectively [27].

Taken together, the resection of NCNNLM from gastroesophageal primaries may offer improved survival rates, although clear selection criteria for patients benefitting from a surgical approach have not yet been defined. However, the above-mentioned studies with metastatic gastroesophageal cancers may not be transferable to EC patients as they mostly consisted of patients with gastric and oesophagogastric junction adenocarcinomas and, if any, a negligible number of distal oesophageal AC. Similarly, there were no patients with oesophageal SCC in these studies (Table 1).

**Table 1:** Studies with metastatic gastroesophageal cancer patients undergoing surgery

Study	Total no. of patients undergoing surgery	No. of patients with synchronous metastases *N* (%)	No. of pat. undergoing hepatectomies for liver metastases *N* (%)	Preop. CTx *N* (%)	Median F/U (mo)	Postop. morbidity (%)	Postop. mortality *N* (%)	Median OS (mo)	3-year OS (%)	5-year OS (%)
Al-Batran 2017	36	36 (100)	n.s	36 (100)	27.5	8.3*	0	31.3**	n.s	n.s
Andreou 2014	47	34 (72.3)	47 (100)	20 (42.6)	76 (1–136)	32	2 (4.3)	18	37	24
Badgwell 2015	82	> 50 (> 61)	n.s	76 (93)	12 (0–168)	50	0	n.s	n.s	25
Carmona-Bayonas 2018	92	> 51 (> 55.4)	25 (27.2)	85 (92)	n.s	19	3 (3.3)	16.7	30.6	n.s
Schmidt 2015	123	123 (100)	23 (20.5)	72 (58.5)	40.1	n.s	4.5 (5)	20***	n.s	n.s

*CTx* chemotherapy, *F/U* follow-up, *OS* overall survival, *n.s.* not stated. Survival times are calculated from the date of metastatic surgery unless otherwise stated

^*^Only morbidities that were reported as serious adverse events

^**^Calculated from the date of arm assignment

^***^Calculated from the date of diagnosis

## Liver resection for oesophageal cancer liver metastases

The literature on patients that underwent surgery for ECLM is scant. The few available studies are of retrospective design with low patient numbers and are significantly biased by patient selection (Table 2). However, there is data that may encourage a more aggressive multimodal treatment approach including surgery in selected patients with stage IVB EC [28–31].

**Table 2:** Studies with patients undergoing liver resection for ECLM

Study	No. of patients undergoing hepatectomy for ECLM	Histopathology *N* (%)	Appearance of metastases	CTx prior to liver resection *N* (%)	Median follow-up (mo)	Median survival (mo)	3-y OS (%)	5-y OS (%)
Adam 2006	20	n.s	n.s	n.s	n.s	16	32	n.s
Huddy 2015	4	3 (75) AC 1 (25) SCC	meta	4 (100)	n.s	10–92	n.s	n.s
Ichida 2013	5	4 (80) SCC 1 (20) n.s	meta	0	n.s	13 (2–70)*	n.s	n.s
Liu 2018	26	26 (100) SCC	meta	n.s	8 (1.0–38.0)	n.s	21.2*	n.s
Seesing 2019	11	9 (81.8) AC 1 (9.1) SCC 1 (9.1) n.s	meta > syn	< 4 (< 21.1)	54	52	55	27
Van Daele 2017	7 (12**)	9 (75) AC 3 (25) SCC	syn	7 (100)	22 (8–50)	22 (8–51)*	n.s	n.s

*ECLM* oesophageal cancer liver metastases, *CTx* chemotherapy, *AC* adenocarcinoma, *SCC* squamous cell carcinoma, *syn* synchronous, *meta* metachronous, *n.s.* not stated. Survival times are calculated from the date of metastatic surgery unless otherwise stated

^*^Calculated from the date of ECLM diagnosis

^**^Out of the total of 12 patients, 7 patients underwent hepatectomy for liver metastases and 5 patients underwent resection of distant lymph node metastases

The authors of a retrospective study from 2016 identified 96 patients with stage IVB EC that were treated with chemotherapy followed by concurrent chemoradiotherapy (CRT) [32]. A subset of 14 patients also received surgery (11 patients for non-regional lymph node metastases, 3 patients for distant organ metastases). The median OS of all patients was 21.0 months. The median OS in the subgroup of patients that received surgery was not reached and a corresponding 5-year OS rate of 50.5% was reported vs 20 months and a 5-year OS rate of 11.7% for those without surgery. However, there was a strong patient selection bias as patients in the group receiving surgery tended to be younger and were less likely to have distant organ metastases.

In 2015, Huddy et al. published a case series that included four patients that underwent liver resection for metachronous liver metastases following a curatively intended oesophagectomy [12]. Two out of the four patients were still alive and without evidence for recurrent disease 22 and 92 months after liver resection. Adam et al. found a median OS of 16 months and a 3-year survival rate of 32% in patients, who underwent resection for synchronous and metachronous ECLM [18]. Similar survival rates were reported by Liu and colleagues, who published a retrospective study with 69 consecutive patients with metachronous, solitary ECLM [33]. A subgroup of 26 patients underwent liver resection, whereas the remaining 43 patients were treated conservatively with chemotherapy and additional local therapies including radiofrequency ablation (*n* = 16), high intensity focused ultrasound (*n* = 12), or microwave ablation (*n* = 15). Patients in the surgical group had significantly higher 1- and 2-year survival rates compared to the non-surgically treated patients (50.8% and 21.2% vs. 31% and 7.1%, *p* = 0.027 and < 0.05, respectively).

A recently published study by Seesing et al. included 34 patients, in whom a resection of gastroesophageal cancer metastases was performed [5]. Of these patients, 19 received a resection of hepatic metastases and 15 a resection of pulmonary metastases. A subgroup analysis of the patients with ECLM (*n* = 11) revealed a median OS of 52 months and a 1-, 3-, and 5-year OS of 91%, 55%, and 27%, respectively. The survival after pulmonary metastasectomy (*n* = 11) was even higher with 1-, 3-, and 5-year OS of 82%, 64%, and 64%, respectively. Similar results were provided by Van Deale et al. in a retrospective study consisting of 12 patients with EC and synchronous liver (*n* = 6) or lymph node metastases (*n* = 5) as well as one patient with liver and lymph node metastases [13]. Ten patients underwent an Ivor-Lewis oesophagectomy with a two-field lymphadenectomy. The metastatic liver lesions were treated synchronously via wedge resection (*n* = 4), radiofrequency ablation (*n* = 1), or microwave ablation (*n* = 1); the five patients with distant lymph node metastases underwent surgical resection. After a median follow-up of 22 months, 50% of the surgically resected patients were still alive with 33% being free of disease recurrence.

Ichida et al. conducted a retrospective study that included 315 patients, who had undergone curatively intended oesophagectomies [11]. During a median follow-up period of 47 months, 138 patients (47%) developed disease recurrence. Five out of 26 patients with hepatic recurrences were treated with hepatic metastasectomy. A trend towards an improved survival in the group receiving surgery vs. no surgery could be observed (median OS 13 vs. 5 months). However, the non-surgically treated patients had either multinodular hepatic recurrence and/or additional extrahepatic recurrence or a poor general performance status, which makes a direct comparison with the surgically treated patients difficult. Nonetheless, there was one patient after hepatic resection, who was still alive and without evidence of recurrent disease 70 months after recurrence detection.

A recently published article reviewed studies and case reports with patients suffering from liver oligometastatic EC [4]. The authors concluded that surgery seems to be the treatment of choice for resectable ECLM, especially for patients with 3 or less lesions. However, the review included only retrospective studies and small case reports/case series. Due to their significant heterogeneity, patient selection bias, small patient numbers, and a lack of defined treatment protocols, these studies may not support such definite conclusions.

Nevertheless, the survival rates provided by Seesing and van Daele following ECLM resection are very encouraging [5, 13]. Therefore, the authors suggest to discuss the option of ECLM resection in MDT meetings, especially for suitable patients with liver oligometastatic disease.

## Factors associated with an improved survival after resection of ECLM

### Preoperative chemotherapy

As most of the studies with patients undergoing ECLM resection included only small patient numbers, the role of preoperative (palliative/neoadjuvant) chemotherapy has not been assessed appropriately. However, there is some evidence that preoperative chemotherapy may positively impact on survival rates, as a response to chemotherapy or a lack of progression was either a positive prognostic marker in surgically treated metastatic gastroesophageal cancer patients or it was used as selection criterion for a surgical approach in ECLM in the first place [12, 13, 24, 25, 27]. However, this could be more accurate for patients with synchronous ECLM that were often treated with simultaneous resection of the primary tumour and the metastasis(-es). Seesing and colleagues demonstrated superior survival rates in patients with predominantly metachronous liver metastases that underwent surgery to a large extent without prior chemotherapy [5]. On the contrary, all five patients in the study by Ichida et al. underwent surgery for metachronous ECLM without prior chemotherapy and had a relatively poor median survival of 13 months postmetastasectomy [11].

Wang et al. investigated the impact of a multimodal treatment approach in stage IVB EC that included surgery of the primary tumour and the metastatic lesion in selected cases [32]. All patients (*n* = 96) received palliative chemotherapy followed by concurrent CRT. Of these, 14 patients also underwent surgery as mentioned above. In a multivariate analysis, the radiographic response of the primary tumour to induction chemotherapy (complete vs incomplete) was associated with an improved OS. A radiologic complete response was defined as a lack of residual SUV after treatment assessed via PET/CT. When concurrent CRT response variables were not included in the multivariate analysis, the receipt of surgery was a significant independent predictor of improved OS and disease-free survival (DFS). Intriguingly, the radiographic response of the primary tumour to induction chemotherapy or concurrent CRT appeared to be of more significance than the response of the metastatic lesions.

Another study from 2015 included 112 patients with metastatic gastric or oesophagogastric junction adenocarcinomas that underwent surgical resection [25]. In the subgroup of patients that received neoadjuvant treatment (*n* = 72), the clinical responders (defined by a decrease of the maximal transversal primary tumour diameter of > 50% measured on CT and a decrease of the endoluminal tumour size of > 75% on endoscopic findings) had a significantly prolonged median survival compared to the non-responders (77.3 months vs 23.5 months; *p* < 0.001). Moreover, the group that did not receive preoperative chemotherapy had a significantly reduced median survival compared to the group, in which a preoperative chemotherapy was administered (11.0 months vs 31.1 months, *p* < 0.001). Therefore, the authors of the study concluded that a primary resection is not appropriate for patients with metastatic gastroesophageal cancer.

Similar findings were provided by Andreou et al., who published a retrospective study with 47 gastroesophageal cancer patients, in whom hepatic resections for mainly synchronous liver metastases were performed [27]. Of these, 32 patients underwent a simultaneous resection of the primary tumour and hepatic metastases. In the multivariate analysis, not undergoing preoperative chemotherapy was significantly associated with poor survival (5-year OS: 9% vs 45%, *p* = 0.005). In the subset of patients receiving preoperative chemotherapy (*n* = 20), the patients responding to the preoperative treatment or with stable disease on radiographic imaging (*n* = 13) had a significantly improved OS compared to those with progressive disease (*n* = 7) (5-year OS rate: 70% vs 0%, *p* = 0.045).

As opposed to the two studies mentioned above, a Dutch study reported superior survival rates in 19 patients with hepatic metastases from gastroesophageal primaries (8 × gastric primary, 11 × oesophageal primary), of which only 4 received neoadjuvant treatment prior to metastasectomy [5]. After resection of the predominantly metachronous liver metastases (17 × deriving from AC, 2 × from SCC), the median OS was 28 months and the 3- and 5-year survival rates were 41% and 31%, respectively. These contrary findings can lead to the conclusion that synchronous and metachronous liver metastases from gastroesophageal primaries have a differing underlying tumour biology and may require different patient selection criteria and treatment approaches.

However, the timing after receipt of preoperative (palliative) chemotherapy may play a pivotal role that needs to be taken into consideration when patients with ECLM are evaluated for surgery as the group around Carmona-Bayonas found that a longer duration of chemotherapy prior to surgery increases mortality (HR 1.04, *p* = 0.009) [24].

### Patient and tumour-related factors

In a study from 2014 on 23 patients with pulmonary, predominantly metachronous metastases from oesophageal SCC, a short disease-free interval (DFI) < 12 months was associated with poor survival (*p* = 0.02) [34]. These results were in keeping with previous study results from 2008 [35]. Liu et al. could show similar results for patients with ECLM [33]. In this study, the outcomes of patients that underwent surgery for metachronous ECLM were compared with a group of non-surgically treated patients. A DFI > 12 months was associated with a significantly improved survival rate in both groups (*p* [both] < 0.05). Seesing and colleagues reported superior survival rates in 11 patients following the resection of mainly metachronous ECLM [5]. The patients included in the study had a DFI between 11 and 27 months, which supports the assumption that patients with a longer DFI may benefit from surgery. Conversely, Ichida et al. reported a median DFI of 6 months (0–14) in patients that underwent ECLM resection [11]. The study could not demonstrate a survival benefit of the surgically treated patients compared to a group that was treated conservatively (median survival 13 vs 5 months, *p* = 0.06).

The differentiation of the tumour may also have prognostic relevance when evaluating patients for ECLM resection. Poorly differentiated primary tumours have been mentioned as negative prognostic markers in metastatic EC [34].

Moreover, a complete resection of all tumour manifestations with overall clear resection margins (R0-resection) was reported to have a significant impact on survival rates in limited metastatic gastroesophageal cancers [25, 27, 32, 36]. Schmidt et al. demonstrated that complete resection of the primary tumour and the metastases in these patients lead to a significantly improved median survival of 29.5 months compared to patients with incomplete resection (R1/2) (*p* = 0.003) [25]. Andreou et al. could show that an R1-resection in patients that underwent hepatectomy for oesophagogastric cancer was associated with a poor OS [27]. Chao and colleagues revealed that scheduled surgery following CRT in patients with oesophageal SCC and distant nodal metastases (nodal M1a/b disease) leads to a survival benefit only in patients, in whom an R0-resection had been achieved (median OS 45 months vs 9.5 months after incomplete resection and 10.5 months in patients receiving definitive CRT, *p* = 0.0013) [36]. Analogously, the two studies with the longest OS following ECLM resection had high rates of R0-resections of about 90% [5, 13].

In the available literature, only patients with a low number of liver metastases/metastatic deposits underwent resection of ECLM. In the two studies with the longest OS after resection of ECLM, 77% (*n* = 20) of the patients had solitary liver metastases, 15% (*n* = 4) had two liver lesions, and only one patient was treated for 3 liver metastases [5, 13]. Although significantly affected by selection bias, these results may indicate that patients with a low metastatic burden could potentially benefit from a surgical approach. Therefore, a thorough preoperative radiographic imaging including CT, MRI, PET-CT, and (endoscopic) ultrasound/CEUS is of utmost importance [37].

Furthermore, the patients’ age and performance status need to be taken into account like in other areas where major surgery is being considered as young and relatively fit patients with stage IVB EC seem to benefit from an aggressive multimodal therapy including surgery [32].

The response to chemotherapy seems to be a significant factor in patients undergoing ECLM resection (this may also hold true for the response to targeted therapies/immunotherapies, although studies referring to this are not available as of yet). Therefore, in patients with a good response to chemotherapy and/or other potentially favourable factors such as a long disease-free interval in metachronous disease, low number of liver metastases, young age, and good overall performance status, ECLM resection should be considered, if an R0 resection status is achievable.

## Discussion

Liver resections for liver oligometastatic EC remain a controversial issue as the available data is scarce and often of poor quality. Nevertheless, the few available studies demonstrated a survival benefit in patients that underwent liver resections in stage IVB EC, if compared to palliative chemotherapy only [3, 13]. In highly selected patients, 5-year survival rates up to 27% have been reported [5]. Even a chance for long-term survival seems possible [11, 12]. However, the main limitation of all the available studies on ECLM resection is that they are significantly biased. The lack of consistent patient selection criteria and treatment protocols disallow any definite conclusions as to whether ECLM resection should be part of the common armamentarium in the treatment of metastatic EC. Nevertheless, although being of limited validity, the available data speaks against the proposition that there is no role for surgery in metastatic EC. Patients with liver oligometastatic EC that responded well to palliative chemotherapy/CRT or with a long DFI > 12 months after primary surgery may benefit from ECLM resection, given a good performance status and the chance of a complete resection of all tumour manifestations [5, 27, 32, 33]. At the same time, the potential intraoperative and/or postoperative complications of liver resections need to be taken into account. In spite of the improvements in surgical techniques and perioperative management, the potential complications accompanying liver resections may further shorten the already limited lifetime of patients with metastatic EC, especially when extended hepatectomies are required in order to achieve an R0-resection status [6]. In patients with liver metastases from gastric and oesophageal cancer, posthepatectomy complications were found to be an independent predictor of poor overall survival [27]. In some cases, the required extent of liver resection is being underestimated in the preoperative radiographic imaging [26]. In accordance with the principle ‘primum non nocere’, a relevant number of planned ECLM resections may have to be abandoned after surgical exploration if the resection of ECLM would require more extended hepatectomies. Alternatively, surgical and interventional approaches such as radiofrequency ablation or microwave ablation can be combined in order to avoid major liver resections [4]. In this context, high-quality imaging (i.e. MRI with liver-specific contrast and diffusion weighting) is of vital concernment in order to develop a thorough multimodal treatment plan in patients with ECLM [37, 38].

A tool similar to the one developed by Blank and colleagues for patients with metastatic gastric and oesophagogastric junction cancer may help to identify patients that could benefit from ECLM resection [39]. The score defines a low-risk group according to tumour differentiation, histopathological response to prior chemotherapy, and (anticipated) resection status (complete vs. incomplete resection). Patients in the low-risk group (*n* = 22) had a significantly improved median survival of 35.3 months and a 3-year OS of 47.6% compared to 12.0 months and a 3-year OS of 14.2% in the high-risk group (*n* = 126).

There is further evidence that may favour surgery in patients with limited metastatic disease from oesophageal AC. The AIO-FLOT 3 trial evaluated the outcomes in patients with limited metastatic gastric or EGJ cancer, who received chemotherapy followed by surgical resection [40]. Out of the 67 patients with limited metastatic disease, 36 (60%) proceeded to surgery. The median OS was 31.3 months for patients who underwent surgery compared to 15.9 months for patients that received a non-surgical treatment. A limitation of these results is the lack of randomization for the patients with limited metastatic disease in the group receiving surgery vs chemotherapy only. To address this issue, the RENAISSANCE (AIO-FLOT 5) Trial as a prospective, multicentre phase III trial is currently recruiting patients with the aim to compare these two groups in a randomized manner [41]. Whether these findings can be adopted for ECLM will require further prospective studies, although a comparable outcome for distal oesophageal adenocarcinoma seems to suggest itself because of its molecular similarity with gastric cancer [42]. However, a transferability of the results to liver metastases deriving from oesophageal SCC is problematic as oesophageal SCCs seem to have a different tumour biology and differences in lymphatic spread compared to adenocarcinomas [42–44]. Therefore, ECLM from SCC and AC should be separately evaluated.

## Conclusion

The available literature is limited and does not facilitate definite conclusions as to whether the resection of ECLM should be standard practice as part of the multimodal treatment concept in metastatic oesophageal cancer. Notwithstanding this, there is enough evidence to justify further studies to identify those select patients with a favourable tumour biology who may benefit from such treatment approaches, especially in the light of emerging immunotherapies.
